# Replication and Transmission of H9N2 Influenza Viruses in Ferrets: Evaluation of Pandemic Potential

**DOI:** 10.1371/journal.pone.0002923

**Published:** 2008-08-13

**Authors:** Hongquan Wan, Erin M. Sorrell, Haichen Song, Md Jaber Hossain, Gloria Ramirez-Nieto, Isabella Monne, James Stevens, Giovanni Cattoli, Ilaria Capua, Li-Mei Chen, Ruben O. Donis, Julia Busch, James C. Paulson, Christy Brockwell, Richard Webby, Jorge Blanco, Mohammad Q. Al-Natour, Daniel R. Perez

**Affiliations:** 1 Department of Veterinary Medicine, University of Maryland, College Park and Virginia-Maryland Regional College of Veterinary Medicine, College Park, Maryland, United States of America; 2 OIE, FAO and National Reference Laboratory for Avian Influenza and Newcastle Disease, Istituto Zooprofilattico Sperimentale delle Venezie, Viale dell'Università, Legnaro, Padova, Italy; 3 Molecular Virology and Vaccines Branch, Influenza Division, Centers for Disease Control and Prevention, Atlanta, Georgia, United States of America; 4 Department of Chemical Physiology, The Scripps Research Institute, La Jolla, California, United States of America; 5 Department of Molecular Biology, The Scripps Research Institute, La Jolla, California, United States of America; 6 Division of Virology, Department of Infectious Diseases, St. Jude Children's Research Hospital, Memphis, Tennessee, United States of America; 7 Virion Systems, Inc., Rockville, Maryland, United States of America; 8 Department of Pathology and Animal Health, Faculty of Veterinary Medicine, Jordan University of Science and Technology, Irbid, Jordan; University of Liverpool, United Kingdom

## Abstract

H9N2 avian influenza A viruses are endemic in poultry of many Eurasian countries and have caused repeated human infections in Asia since 1998. To evaluate the potential threat of H9N2 viruses to humans, we investigated the replication and transmission efficiency of H9N2 viruses in the ferret model. Five wild-type (WT) H9N2 viruses, isolated from different avian species from 1988 through 2003, were tested in vivo and found to replicate in ferrets. However these viruses achieved mild peak viral titers in nasal washes when compared to those observed with a human H3N2 virus. Two of these H9N2 viruses transmitted to direct contact ferrets, however no aerosol transmission was detected in the virus displaying the most efficient direct contact transmission. A leucine (Leu) residue at amino acid position 226 in the hemagglutinin (HA) receptor-binding site (RBS), responsible for human virus-like receptor specificity, was found to be important for the transmission of the H9N2 viruses in ferrets. In addition, an H9N2 avian-human reassortant virus, which contains the surface glycoprotein genes from an H9N2 virus and the six internal genes of a human H3N2 virus, showed enhanced replication and efficient transmission to direct contacts. Although no aerosol transmission was observed, the virus replicated in multiple respiratory tissues and induced clinical signs similar to those observed with the parental human H3N2 virus. Our results suggest that the establishment and prevalence of H9N2 viruses in poultry pose a significant threat for humans.

## Introduction

Influenza A viruses of the H9N2 subtype have become highly prevalent in poultry in many countries, and although these viruses generally cause only mild to moderate disease, they have been associated with severe morbidity and mortality in poultry as a result of co-infection with other pathogens [1], [2]. Antigenic and genetic analyses of H9N2 viruses isolated during the last two decades indicate that these viruses are extensively evolving and have reassorted with other avian influenza viruses to generate multiple novel genotypes [3]–[7]. Prior to 1990, H9N2 viruses were mainly detected in avian species in North America and “healthy” ducks during surveillance in Southeast China [1]. In 1988, the isolation of an H9N2 virus from Japanese quail in Southern China was the first recorded land-based poultry case of H9N2 in Asia [8], [9]. By 1997, H9N2 viruses had been isolated in multiple avian species throughout Asia, the Middle East, Europe and Africa [10]–[13]. H9N2 viruses have also been reported in swine [5], [14]–[16], the proposed “mixing vessel” for the genesis of potentially pandemic influenza viruses. A significant proportion of H9N2 field isolates have acquired human virus-like receptor specificity, preferentially binding α2-6 linked sialic acid (SAα2-6) receptors, in contrast to the classic avian virus-like receptor specificity that preferentially binds α2-3 linked sialic acid (SAα2-3) receptors [17]–[19]. Interestingly, a few of the H9N2 viruses that recognize SAα2-6 receptors have transmitted directly to humans, causing mild flu-like illness and the consequent fear that they may become pandemic [20]–[23]. In addition, some investigations suggest that H9N2 viruses may have contributed to the genetic and geographic diversity of H5N1 viruses [22], [24]. These studies highlight the necessity for more comprehensive surveillance and further evaluation of H9N2 viruses with proper *in vitro* and *in vivo* models.

Several species of poultry (including chickens, quail, and pheasants) and mammals (such as mice and hamsters) have been used to study H9N2 viruses [3], [17], [25]–[30]. However, none of these models truly reflect the transmission of influenza in humans. Pigs have also been used to study H9N2 viruses [17], however the requirement for proper facilities may limit the use of pigs in influenza studies. Ferrets (*Mustela putorius furo*), which have been shown to physiologically resemble humans in terms of the expression and distribution of sialic acid receptors in the respiratory tract, serve as an ideal model to evaluate the potential risk of H9N2 viruses to public health. The ferret upper respiratory tract expresses predominantly SAα2-6 receptors [31], very similar to that found in human airway epithelium [32]–[34]. The virulence, pathology, and immune response to influenza virus infections in ferrets are similar to those in humans [31]. In recent years, ferrets have been used to evaluate the replication and transmission of the recovered 1918 H1N1 viruses, recent H5N1 viruses and various H5N1-H3N2 reassortants, as well as viruses of the H7 subtype [35]–[39]. The replication, virulence and transmission phenotypes of these viruses in ferrets correlated well with those in humans. To date, there is little information regarding the pathogenicity and/or transmission of H9N2 viruses in the ferret model, particularly those isolated in recent years. In this study, we examined the replication and transmission of H9N2 viruses in ferrets with a particular focus on the role amino acid Leu226 of HA plays in replication and transmission. We have found that Leu226-containing viruses are more likely to transmit in ferrets although this receptor specificity feature alone cannot support aerosol transmission.

## Results

### Replication and direct contact transmission of avian H9N2 viruses in ferrets

Eight WT H9N2 viruses isolated during the period of 1977 to 2003 were used in this study (Table 1). Six of these viruses, Dk/HK/149/77, Dk/HK/Y280/97, Ck/HK/SF3/99, Qa/HK/NT16/99, Hu/HK/1073/99, and Ck/Jordan/554/03 were field isolates. The remaining two, RGWF10 and RGQa88, were WT viruses rescued using reverse genetics. Sequencing analysis revealed that the HA of RGWF10, Dk/HK/Y280/97 and Ck/HK/SF3/99 contain Leu226 at the RBS, whereas RGQa88 and Ck/Jordan/554/03 contain glutamine (Gln) at this position [40].

**Table 1:** Wild-type influenza viruses used in this study

Virus	Subtype	Abbreviated name	Residue at 226
A/Duck/Hong Kong/149/77	H9N2	Dk/HK/149/77	Gln
A/Quail/Hong Kong/A28945/88	H9N2	RGQa88^a^	Gln
A/Duck/Hong Kong/Y280/97	H9N2	Dk/HK/Y280/97	Leu
A/Chicken/Hong Kong/SF3/99	H9N2	Ck/HK/SF3/99	Leu
A/Guinea fowl/Hong Kong/WF10/99	H9N2	RGWF10^a^	Leu
A/Quail/Hong Kong/NT16/99	H9N2	Qa/HK/NT16/99	Leu
A/Hong Kong/1073/1999	H9N2	Hu/HK/1073/99	Leu
A/Chicken/Jordan/554/03	H9N2	Ck/Jordan/554/03	Gln
A/Mallard/Potsdam/178-4/83	H2N2	Mal/Potsdam/83	Gln
A/Memphis/14/98	H3N2	RGMemphis98^a^	Leu

a RGWF10, RGQa88 and RGMemphis98 are WT viruses generated by reverse genetics.

To evaluate the replication and transmission of avian H9N2 viruses, we first determined whether H9N2 viruses could establish significant infections in the ferret model and whether these viruses could be transmitted to direct contact ferrets. Five of these viruses were used in replication and transmission studies in ferrets. For each virus tested, two ferrets were directly infected with 10^6^ TCID_50_ (median tissue culture infectious dose) of virus (in the case of RGWF10, 3 ferrets were inoculated). At 24 h post-inoculation (pi), a direct contact ferret was introduced into the same cage as each infected ferret. Ferrets were monitored as described in Materials and Methods. No overt signs of disease, including sneezing, were observed in any of the inoculated ferrets with any of the five WT H9N2 viruses used. However, lethargy and anorexia were noted in some cases, usually lasting 2 to 3 days. The inoculated ferrets experienced slight body weight loss (average <3%) (Table 2). Transient elevation of body temperature (maximum elevation between 0.7 to 2.1°C) was detected in all RGWF10-infected ferrets and in at least one ferret from each of the remaining H9N2 viruses. Temperatures were highest between 2 to 3 days pi, when a majority of the viruses were at peak shedding in nasal washes.

**Table 2:** Clinical signs, virus replication and seroconversion in inoculated ferrets.

Virus	Inoculated ferrets		
	Weight loss (%)^a^	Sneezing (Day of onset)	Serum (HI titer)^b^
RGWF10^c^	1.8±0.57	0/3	1280, 1280, 1280
RGWF10	2.3^d^	0/2	2560, 2560
Dk/HK/Y280/97	1.55±0.35	0/2	1280, 1280
RGQa88	2.8 d	0/2	1280, 1280
Ck/HK/SF3/99	1.55±0.05	0/2	1280, 1280
Ck/Jordan/554/03	1.9±2.1	0/2	640, 640
RGMemphis98	6.54±0.13	2/2 (3, 3)	5120, 2560
2WF10:6M98	5.1±0.85	2/2 (2, 2)	2560, 2560

a Average body weight loss±SD is shown.

b Homologous virus was used in the HI assays to detect anti-H9 antibodies.

c For the RGWF10 virus, two separate experiments were performed.

d Only one ferret lost body weight.

**Figure 1:**
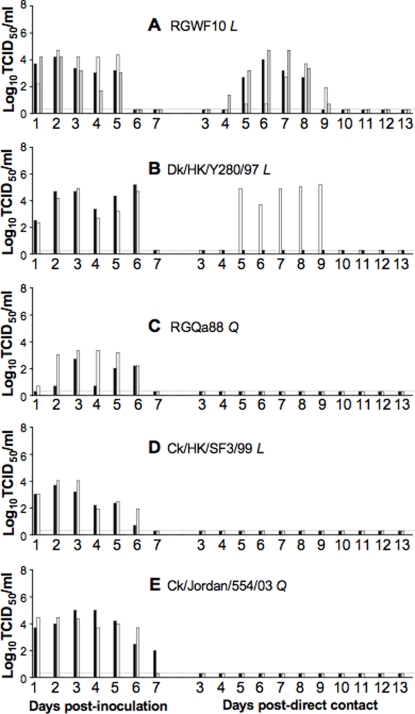
Replication and direct contact transmission of H9N2 viruses. Ferrets were inoculated intranasally (i.n.) with 10^6^ TCID_50_ of H9N2 viruses RGWF10 (A), Dk/HK/Y280/97 (B), RGQa88 (C), Ck/HK/SF3/99 (D), and Ck/Jordan/554/03 (E). Twenty-four hours later, one naïve ferret (direct contact) was added to the same cage as each of the infected ferrets. Nasal washes were collected daily and were titrated in MDCK cells. Black, white and gray bars represent individual ferrets sampled and the amount of viral shedding at different days pi. The titers are expressed as log_10_ values of TCID_50_/ml with the limit of detection at 0.699 log_10_TCID_50_/ml. The dotted line was arbitrarily set at <0.3 log_10_TCID_50_/ml in order to represent samples below the detection limit. *L* and *Q* correspond to Leu226 and Gln226, respectively in the HA RBS.

Virus was detected in the nasal washes from all of the inoculated ferrets, with peak titers ranging from 2.7 to 5.2 log_10_TCID_50_/ml (Fig. 1). The highest peak titer was achieved in the Dk/HK/Y280/97 group while the lowest was in the RGQa88 group. For the RGWF10 group, virus was detected in all the inoculated animals from 1 to 5 days pi and was transmitted to all direct contact ferrets, as demonstrated by the detection of virus in nasal washes using FLU DETECT™ Antigen Capture Test Strip (data not shown), and viral titration (Fig. 1A). The direct contact animals began to shed detectable levels of virus by day 4 and 5 post-contact (pc), and each shed virus for 4 to 6 days, with peak titers comparable to those found in the inoculated ferrets (Fig. 1A). Anti-H9 antibodies were detected in all ferrets, with hemagglutination inhibition (HI) titers of 1280 in the inoculated ferrets, and 320 to 640 in the contact ferrets. Virus was detected in both Dk/HK/Y280/97-inoculated ferrets for 6 days and transmitted to 1 of the 2 direct contacts, which shed virus for 5 days (Fig. 1B). In the RGWF10 and Dk/HK/Y280/97 groups, the viral positive contacts exhibited lethargy, anorexia, weight loss and elevated temperature similar to the inoculated ferrets. The RGQa88, Ck/HK/SF3/99 and Ck/Jordan/554/03 groups shed viruses for up to 7 days (Figs. 1C, D, and E), and developed high titers (640–1280) of H9 antibodies (Table 2). However, neither virus shedding nor seroconversion was detected in any of the contact ferrets (Figs. 1C, D, E and Table 3), reflecting the lack of direct contact transmission. These results suggest that the ferret model is able to recapitulate the infection of H9N2 viruses as observed in humans and pigs. Our findings suggest that the ferret represents a good animal model to study the potential changes that could lead to efficient transmission of avian H9N2 viruses in humans.

**Table 3:** Clinical signs, virus replication and seroconversion in direct and aerosol contact ferrets.

Virus	Direct contact ferrets			Aerosol contact ferrets		
	Virus detected in nasal wash^a^	Sneezing (Day of onset)	Serum (HI titer) b	Virus detected in nasal wash^a^	Sneezing (Day of onset)	Serum (HI titer) b
RGWF10^c^	3/3	0/3	320, 320, 640	ND^d^	ND	ND
RGWF10	2/2	0/2	640, 1280	0/2	0/2	<10, <10
Dk/HK/Y280/97	1/2	0/2	640, <10	ND	ND	ND
RGQa88	0/2	0/2	<10, <10	ND	ND	ND
Ck/HK/SF3/99	0/2	0/2	<10, <10	ND	ND	ND
Ck/Jordan/554/03	0/2	0/2	<10, <10	ND	ND	ND
RGMemphis98	2/2	2/2 (4, 4)	5120, 5120	2/2	2/2 (8,10)	2560, 5120
2WF10:6M98	2/2	2/2 (4, 5)	1280, 1280	0/2	0/2	<10, <10

a Virus in nasal washes was analyzed using FLU DETECT™ Antigen Capture Test Strip (Synbiotics Corp.) and titrated by TCID_50_.

b Homologous virus was used in the HI assays to detect anti-H9 antibodies.

c For the RGWF10 virus, two separate experiments were performed.

d ND, not done.

### Lack of aerosol transmission in the ferret model

**Figure 2:**
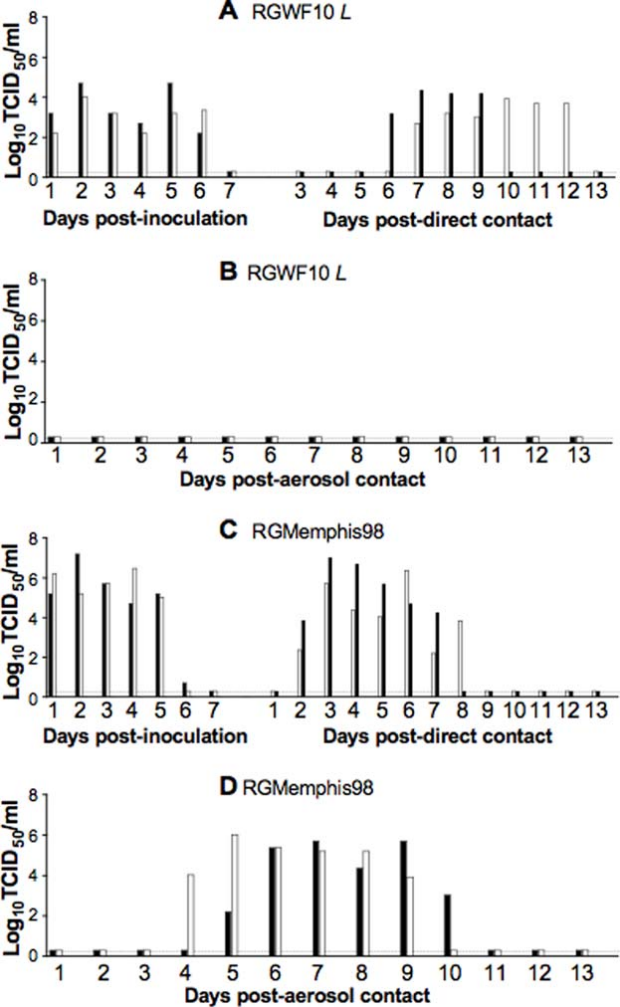
Aerosol transmission of H9N2 and H3N2 viruses. Ferrets were inoculated i.n. with 10^6^ TCID_50_ of RGWF10 or RGMemphis98 virus. Twenty-four hours later, one naïve ferret was added to each infected ferret to serve as direct contact, and another ferret was placed into an adjacent cage separated by a wire mesh to serve as aerosol contact. Nasal washes were collected daily and were titrated in MDCK cells. (A) RGWF10 infected and direct contacts. (B) RGWF10 aerosol contacts. (C) RGMemphis98 inoculated and direct contacts. (D) RGMemphis98 aerosol contacts.

We tested next whether the RGWF10 virus could be transmitted by aerosol. The RGWF10 virus was selected due to its efficient transmission to direct contacts and its lineage; belonging to the A/Quail/Hong Kong/G1/97-like viruses, which closely resembles the virus isolated from the first human index case of H9N2 infection in 1999 [22], [23]. Two ferrets were inoculated with RGWF10 virus at a dose of 10^6^ TCID_50_ and 24 h later, the direct contact and aerosol contact ferrets were introduced, as previously described. No overt clinical signs of disease were observed in the inoculated ferrets; however, they displayed slight weight loss and transient elevation of body temperature, as noted with the initial study. As shown in Fig. 2A, viral shedding was detected in both inoculated and direct contact ferrets. By day 14 pi or pc, both the inoculated and direct contacts developed high titers of anti-H9 antibodies (2560 for the inoculated ferrets, 640 and 1280 for direct contacts). However, no viral shedding or seroconversion was detected in the aerosol contacts (Fig. 2B and Table 3), indicating the lack of aerosol transmission of RGWF10.

To validate our system of detecting aerosol transmission, we undertook transmission studies performed with prototypic human and avian viruses, A/Memphis/14/98 (H3N2) (RGMemphis98), and A/Mallard/Potsdam/83 (H2N2) (Mal/Potsdam/83). High titers of virus were detected in all ferrets in the RGMemphis98 group: inoculated, direct- and aerosol-contacts (Figs. 2C and D). The aerosol contacts began shedding virus by day 4 and 5 pc, respectively, and both shed virus for up to 6 days. All ferrets showed clinical signs including sneezing and developed high antibody titers against RGMemphis98 (Tables 2 and 3). For the Mal/Potsdam/83 group, the virus was shed from inoculated ferrets for 3 to 4 days (data not shown). No viral shedding or seroconversion was detected in any of the direct or aerosol contact ferrets. Taken together, these data indicate that although some H9N2 viruses can transmit to direct contacts, they lack successful aerosol transmission.

### A Gln226Leu mutation in the RBS of HAs of H9N2 viruses improves direct-contact transmission in ferrets

We have previously shown that residue 226 in the RBS of H9N2 HAs is important for viral growth in human airway epithelial (HAE) cells *in vitro* [40]. Viruses containing Leu226 in the HA RBS grew to significantly higher titers in HAE cultures than viruses with Gln226 [40]. In order to determine the role of residue 226 *in vivo*, we initially examined whether the Leu226Gln mutation in a natural Leu226-containing H9N2 virus would affect viral replication and transmission in ferrets.

**Figure 3:**
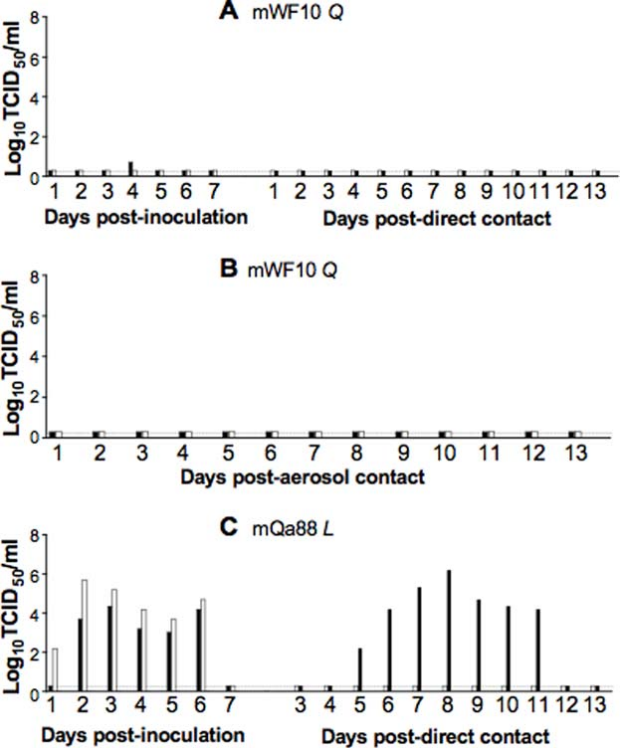
Replication and transmission of mutant H9N2 viruses. Ferrets were inoculated i.n. with 10^6^ TCID_50_ of mWF10 (Gln226) or mQa88 (Leu226) virus. Twenty-four hours later, contact ferrets were introduced as described above. Nasal washes were collected daily and were titrated by TCID_50_. (A) mWF10 infected and direct contacts. (B) mWF10 aerosol contacts. (C) mQa88 infected and direct contacts. *L* and *Q* correspond to Leu226 and Gln226, respectively in the HA RBS.

Using site-directed mutagenesis we altered Leu226 in the HA RBS of RGWF10 to Gln, creating the mutant WF10 (mWF10). The mWF10 virus grew as efficiently as RGWF10 in eggs and MDCK cells [40]. To test the replication and transmission of mWF10, we inoculated two ferrets with 10^6^ TCID_50_ and introduced direct and aerosol contact ferrets at 24 h pi. Trace amounts of viral shedding were detected in one of the two inoculated ferrets and neither showed signs of disease. No virus was recovered in either the direct or aerosol contact ferrets (Figs. 3A and B). Both of the inoculated ferrets seroconverted with low anti-H9 titers (40 and 80, respectively). However, no seroconversion was detected in the contact ferrets. Thus, mutation from Leu to Gln at HA position 226 of H9N2 viruses drastically reduced virus replication and completely abolished transmission to direct contacts.

To determine whether the change from Gln to Leu alone in a natural Gln226-containing virus allows for transmission, a Gln226Leu mutation was introduced in the HA RBS of RGQa88 and the mutant, mQa88, was recovered. The mQa88 displayed human virus-like cellular tropism in HAE cultures [40]. To determine transmissibility, two ferrets were inoculated with 10^6^ TCID_50_ and two direct contact ferrets were introduced at 24 h pi. As shown in Fig. 3C, the inoculated ferrets shed virus with peak titers significantly higher (approximately >1.5-log_10_) than the WT virus RGQa88 (Fig. 1C). In addition, the mQa88 virus was transmitted to one of two direct contacts and virus was shed for 7 consecutive days. Therefore, a single amino acid change of Gln to Leu at amino acid 226 in the HA RBS enhanced both replication and transmission of mQa88 virus in ferrets.

### Analysis of H9N2 virus receptor specificity by glycan microarray

The importance of Leu226 in replication and transmission of influenza viruses in ferrets is presumably related to the acquisition of specificity for human-type SAα2-6 receptors. Using various assays, previous reports have documented early H9N2 viruses with Gln226 to exhibit dual binding to sialosides with the NeuAcα2-3 and NeuAcα2-6 linkages, while more recent avian and human isolates with Leu226 exhibit preferential specificity for NeuAcα2-6 linkages [4], [17]–[19], [40]. The assays used in these studies gave limited specificity information besides the terminal sialic acid linkage. To further characterize and understand the transmissibility of these H9N2 viruses, we explored their receptor specificity using glycan microarray technology to survey more than 100 sialoglycans simultaneously [41], [42].

**Figure 4:**
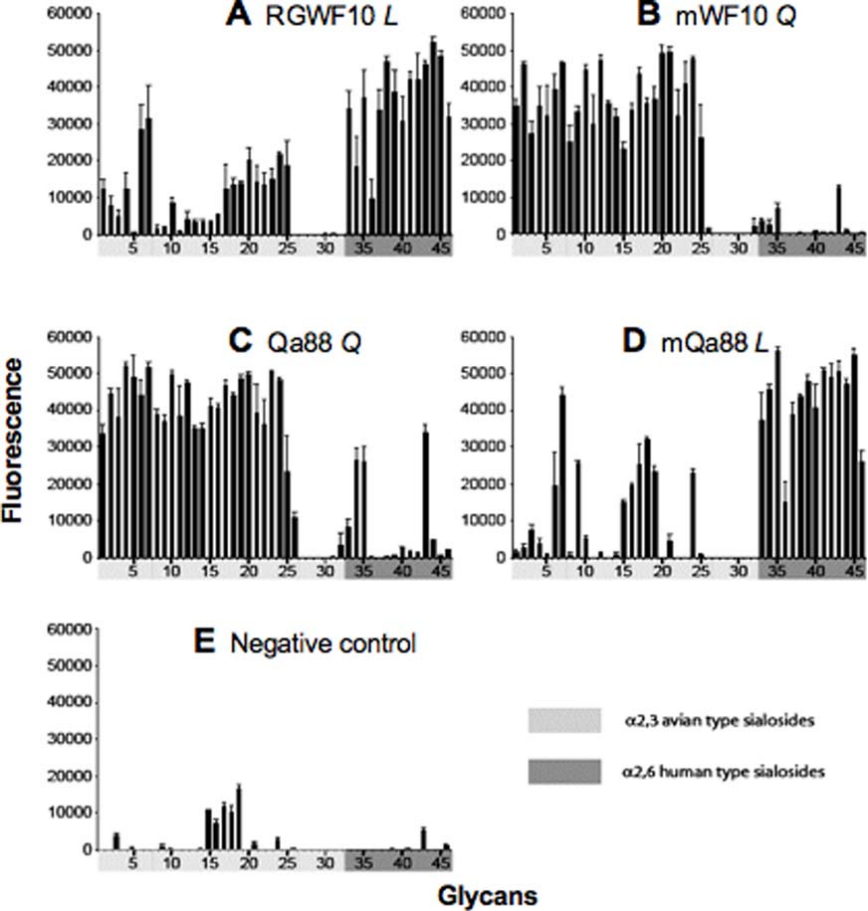
Effect of Leu226 or Gln226 mutations on receptor specificity of H9N2 viruses. The importance of amino acid 226 of HA in receptor specificity was confirmed by site-directed mutagenesis followed by glycan microarray analysis. Viruses with a natural Leu226 (A: RGWF10 *L*) or Gln226Leu mutation (D: mQa88 *L* ) bind to human-type α2-6 sialosides (glycans 33 to 46), whereas viruses with Leu226Gln mutation (B: mWF10 *Q*) or natural Gln226 (C: RGQa88 *Q*) bind to avian-type α2-3 sialosides (glycans 1 to 32). Viruses were analyzed at hemagglutination titers of 128 per 50 µl. Allantoic fluid was used as negative control (E). Glycans 1–32 are avian-type α2-3 sialosides (light gray) and 33–46 are human-type α2-6 sialosides (dark gray). *L* and *Q* correspond to Leu226 and Gln226, respectively in the HA RBS. The complete structures of each sialoside are available upon request.

The WT viruses RGWF10 (Leu226), RGQa88 (Gln226), and their corresponding mutant viruses mWF10 (Gln226), mQa88 (Leu226), were analyzed for their receptor glycan preference. Results for these H9N2 viruses towards sialosides with the terminal NeuAcα2-3Gal linkage (33 glycans) and NeuAcα2-6 linkage (13 glycans) are shown in Fig. 4. The Leu226-containing viruses (RGWF10 and mQa88) exhibited a strong preference for binding human-type α2-6 sialosides and minimal binding to α2-3 sialosides (Figs. 4A and D), while the Gln226-containing viruses (mWF10 and RGQa88) exhibited strong preferential binding to mainly avian-type α2-3 receptors (Figs. 4B and C). These data clearly demonstrate that a Leu226 in the HAs of H9N2 viruses confers human virus-like receptor specificity, while the presence of Gln226 in the HAs reduces binding to α2-6 sialosides and shifts the preference to α2-3 sialosides.

**Figure 5:**
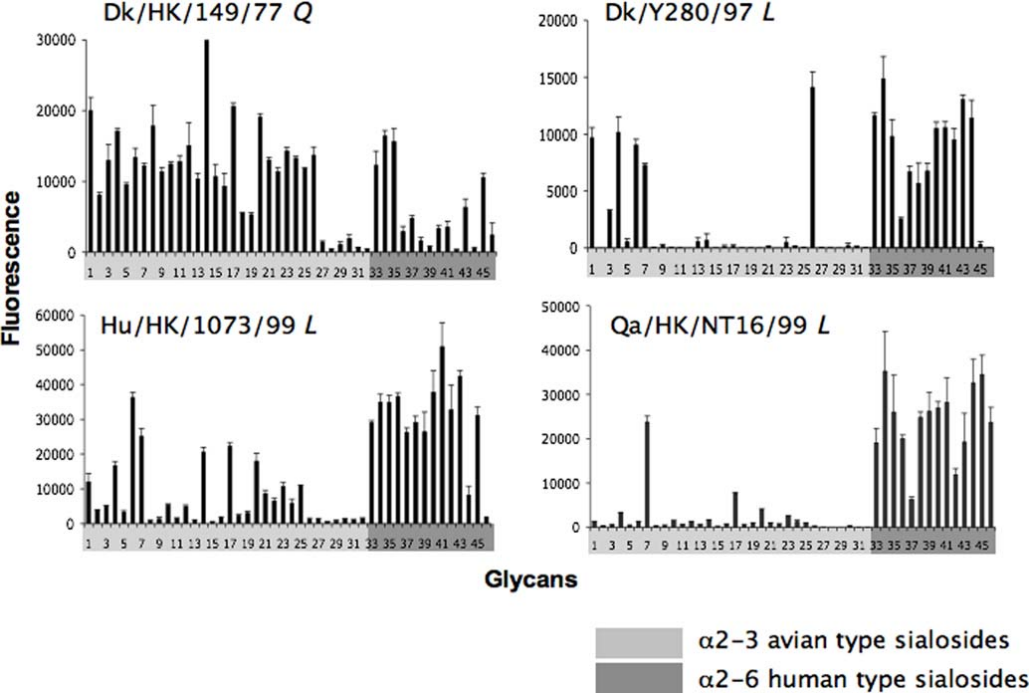
Receptor specificity of other wild type H9N2 isolates. Other selected H9N2 isolates from 1977 to 1999 were assessed on the glycan microarray as previously described [49]. Shown are results for Dk/HK/149/77, an isolate with Gln226 (*Q*), and three later isolates with Leu226 (*L*) Dk/Y280/97, Hu/HK/1073/99, and Qa/HK/NT16/99. Viruses were analyzed at a hemagglutination titer of 256 or 128 (Hu/HK/1073/99). *L* and *Q* correspond to Leu226 and Gln226, respectively in the HA RBS.

Glycan array analysis of other WT viruses confirms and extends the importance of this single amino acid on receptor specificity of H9N2 viruses (Fig 5). Dk/HK/149/77 with a Gln226 exhibited dual specificity with preference for α2-3 sialosides, while the more recent viruses with Leu226, comprising two avian isolates and one human isolate (Dk/HK/Y280/97,Qa/HK/NT16/99 and Hu/HK/1073/99), exhibited broad preference for α2-6 sialosides (although limited binding to α2-3 sialosides was also evident for all three viruses with no clear pattern). Interestingly, although the specificity of RGWF10 and the Leu226 variant of RGQa88 virus (mQa88) was associated with more residual α2-3 specificity than some of the other WT viruses with Leu226 (Fig. 5), they exhibited significantly increased replication and contact transmission in ferrets. Thus, taken together, these results strongly suggest that a Leu residue at position 226 of the HA RBS selects for human virus-like receptor specificity that enhances replication efficiency and direct contact transmission of avian H9N2 viruses in the ferret model.

### Enhanced replication and transmission of an H9N2 avian-human reassortant virus in ferrets

**Figure 6:**
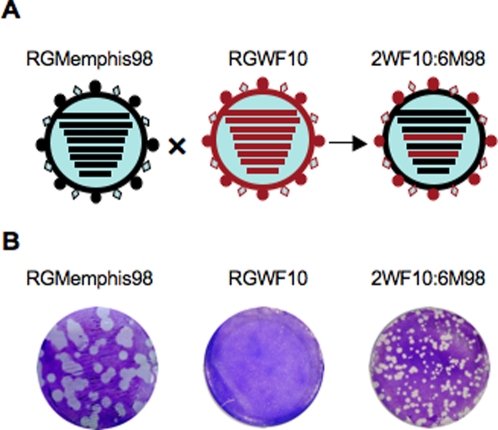
Recovery and plaque assay of an H9N2 avian-human reassortant virus. (A) Diagram outlining gene segment exchange to create the reassortant virus. (B) Plaque morphology of the parental H3N2 virus RGMemphis98 (left), the parental H9N2 virus RGWF10 (center) and the 2WF10:6M98 reassortant virus (right).

Two previous human influenza pandemics, in 1957 (H2N2) and in 1968 (H3N2), were the result of reassortment between low pathogenic avian influenza viruses and circulating human viruses [43], [44]. Due to the multiple introductions of avian H9N2 viruses into the human population in the last decade, we wanted to determine whether an H9N2 avian-human reassortment would enhance the transmissibility of Leu226-containing H9N2 strains. We recovered an H9N2 avian-human reassortant virus, 2WF10:6M98, containing the HA and NA genes of A/Guinea fowl/Hong Kong/WF10/99 (H9N2) and the six internal genes from A/Memphis/14/98 (H3N2), using reverse genetics (Fig. 6A). The reassortant virus grew efficiently in MDCK cells, reaching a titer of 8.7 log_10_TCID_50_/ml, comparable to the titer of the parental H3N2 virus (8.4 log_10_TCID_50_/ml) and higher than the parental H9N2 virus (7.2 log_10_TCID_50_/ml), indicating good compatibility of the gene constellation for this reassortant virus. We also analyzed the growth phenotype of these viruses by plaque assay. We observed that the reassortant 2WF10:6M98 virus and the WT RGMemphis98 virus formed large, clear plaques, while the WT RGWF10 virus only produced pinpoint, less defined plaques (Fig. 6B).

**Figure 7:**
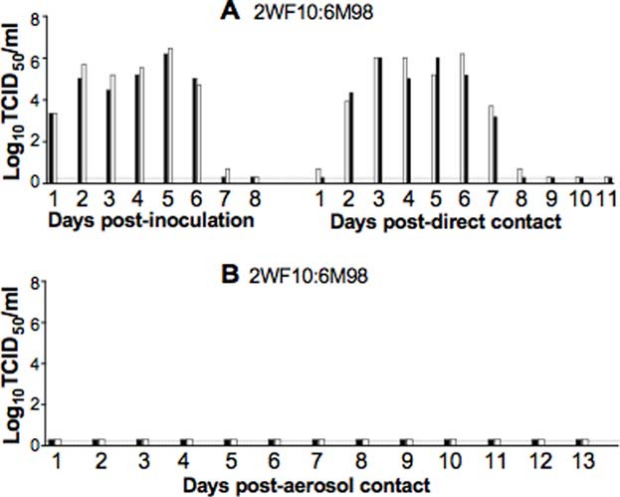
Replication and transmission of the H9N2 avian-human reassortant virus. Ferrets were inoculated i.n. with 10^6^ TCID_50_ of 2WF10:6M98 virus. Twenty-four hours later, the direct and aerosol contacts were placed in the cages as described above. Nasal washes were collected daily and were titrated by TCID_50_. (A) 2WF10:6M98 virus inoculated and direct contacts. (B) 2WF10:6M98 virus aerosol contacts.

The replication and transmission of the 2WF10:6M98 reassortant virus was then investigated. Ferrets were inoculated with 10^6^ TCID_50_ of 2WF10:6M98 and the direct and aerosol contacts were introduced at 24 h pi. As shown in Fig. 7, viral shedding was detected in both inoculated ferrets and direct contacts, with contact ferrets shedding virus on day 2 pc, similar to that observed for WT RGMemphis98 virus. The peak viral titers (>6.0 log_10_TCID_50_/ml) in nasal washes from both inoculated and direct contacts were higher than those from the RGWF10 group (<5.0 log_10_TCID_50_/ml). However, no viral shedding was detected in the aerosol contacts. In addition, the inoculated ferrets and direct contacts developed signs of disease, characterized by lethargy, anorexia and sneezing, similar to those found in RGMemphis98-infected ferrets. The 2WF10:6M98-infected ferrets showed body weight loss (average >5.0%) more than that found in RGWF10-infected animals (Table 2). High antibody titers against H9 were detected in both inoculated and direct contact ferrets, but not in the aerosol contacts (Tables 2 and 3). These results demonstrate that reassortment with a human H3N2 virus enhanced viral shedding and transmission of the H9N2 virus to direct contacts. However, the reassortant virus still lacks the ability to transmit to aerosol contacts.

### Increased pathology and tissue tropism in ferrets of the H9N2 avian-human reasssortant virus

**Figure 8:**
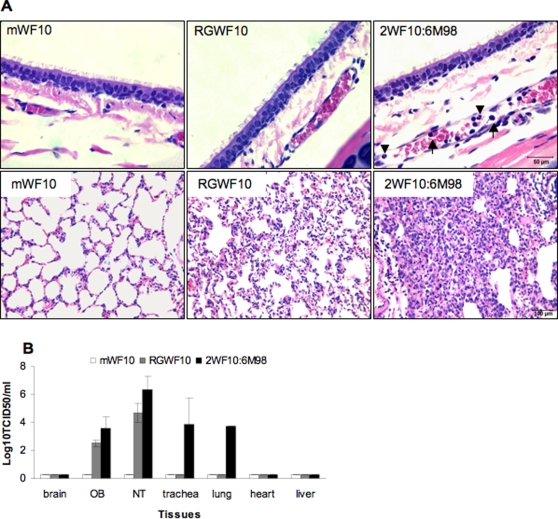
Histopathology and virus distribution of H9N2 viruses in ferrets. Two ferrets were inoculated i.n. with 10^6^ TCID_50_ for each virus: mWF10, RGWF10 or 2WF10:6M98. At day 4 p.i, ferrets were euthanized and the tracheas and lungs were harvested for histological analysis. (A) Histopathological findings in the respiratory tract. Upper panel, tracheas: note the margination of neutrophils (▾) and mononuclear cells (↑) in a small vein in the 2WF10:6M98-infected trachea. Lower panel, lungs: note the severe inflammatory infiltration in the 2WF10:6M98-infected lung. (B) Tissue tropism in organs collected from ferrets inoculated with mWF10, RGWF10, or 2WF10:6M98 virus. OB, olfactory bulb. NT, nasal turbinate.

We further compared the 2WF10:6M98 virus to its parental RGWF10 for histopathology, focusing mainly on the respiratory tissues. We included the replication defective mWF10 as a Gln226-containing virus for comparison. Histological examination of the tissues collected at 4 days pi revealed that the 2WF10:6M98 reassortant virus induced more severe lesions in the lungs. Evident alveolar edema and severe infiltration of inflammatory cells, including mononuclear cells, lymphocytes and neutrophils, were observed (Fig. 8A). The lungs from RGWF10-inoculated ferrets showed only mild lesions, characterized by slight thickening of alveolar septi. Focal alveolar edema was also noted in the lungs. However, the lungs from mWF10-infected ferrets did not show significant pathological changes. In general, the pathology of the tracheas for each virus was less severe than the lungs. Marked margination of neutrophils and mononuclear cells was observed in small blood vessels in the *lamina propria* of the tracheas from the 2WF10:6M98-infected ferrets, while no lesions were observed in tracheas from either the RGWF10 or mWF10 virus-infected animals.

The tissue tropism of the resassortant 2WF10:6M98 virus was also examined and compared to those of RGWF10 and mWF10. On day 4 pi, virus was recovered in multiple tissues from ferrets infected with 2WF10:6M98, including the olfactory bulb, nasal turbinate, trachea and lung. RGWF10 was detected only in the olfactory bulb and nasal turbinate (albeit to lower titers than 2WF10:6M98), while mWF10 was not recovered in any of the tissues examined (Fig. 8B). These results indicate that the H9N2 avian-human reassortant is more virulent for ferrets and has a broader tissue tropism than the parental WT H9N2 virus.

## Discussion

H9N2 viruses are prevalent in avian species in various parts of the world [6], [7], [10]. Several human cases of H9N2 infection have been recorded since 1998 [20]–[23]. The recurring presence of H9N2 infections in humans has raised concerns about the possibility of H9N2 viruses evolving into pandemic strains. Therefore, it is crucial to evaluate the potential pandemic threat posed by H9N2 viruses using appropriate *in vitro* and *in vivo* models. In this study, the replication and transmission of a panel of avian H9N2 viruses, isolated during the last two decades (from 1988 to 2003), were evaluated in the ferret model. Ferrets inoculated with these H9N2 strains shed moderate levels of virus in the nasal washes when compared to a typical human H3N2 strain. Some of the inoculated ferrets showed transient lethargy and temporary body temperature elevation, yet no overt signs of disease (sneezing and/or nasal discharge) were observed. These observations are consistent with the benign nature of H9N2 infection in humans. In contrast to the clinically mild H9N2 infections, ferrets infected with a human H3N2 virus developed clinical signs including sneezing and body weight loss (>6%). By placing two naïve ferrets, one into the same cage as the inoculated ferret and a second into an adjacent cage separated by a wire mesh, we created direct and aerosol transmission models to mimic potential, natural routes of transmission. Our data indicated that 2 out of the 5 WT H9N2 viruses tested were able to transmit from the inoculated to the direct contact ferrets. The infected contacts showed similar levels of viral shedding, body weight loss and serum antibody titers as their inoculated counterparts. Our results suggest that ferrets, which have been used in the studies of influenza viruses of other subtypes, can serve as a useful model for the studies of avian H9N2 viruses, particularly for the evaluation of H9N2 replication and transmission in mammals.

To date, the H9N2 viruses isolated from humans have been considered to be of avian origin [20], although the exact mechanism of transmission of H9N2 viruses from poultry to humans remains to be fully elucidated. It is notable that the human virus-like receptor specificity, related to the presence of Leu226 in the HA appears to be critical for transmissibility of H9N2 viruses to mammals, as revealed by our site-directed mutagenesis studies. Our study revealed that a Leu to Gln mutation of amino acid 226 in the HA RBS severely impaired replication and completely abolished transmission of an H9N2 virus to direct contact ferrets. Restoring a Leu226 into the RBS transformed a nontranmissible virus into one that transmits by direct contact. It is likely that the Leu226-containing viruses are more efficient than Gln226-containing viruses in binding and replicating in ferret airway epithelium because of the rich presence of SAα2-6 receptors. Our glycan data clearly shows a dramatic contrast in the glycan binding pattern of Leu226 vs Gln226 viruses, which could in part explain the transmissibility phenotype of the former. Consistent with this concept, we and others have previously shown that the presence of Leu226 in the HA correlated with enhanced replication of influenza viruses in an *in vitro* HAE model, especially in HAE cells that express mainly SAα2-6 receptors [40], [45]. These observations provide valuable clues for understanding why avian H9N2 viruses carrying the Leu226 signature in the HA can cross the species barrier and cause infections in humans.

Unlike the H5N1 avian-human reassortant viruses that lack transmissibility in the ferret model [37], our H9N2 avian-human reassortant virus replicated in ferrets more efficiently than the parental H9N2 virus (∼2-log_10_ higher peak viral titers in nasal washes), transmitted efficiently among direct contact ferrets, and induced clinical signs of disease. Our *in vitro* study also showed that the reassortant virus induced larger and more defined plaques in MDCK cells than WT H9N2 viruses. Furthermore the reassortant H9N2 avian-human virus showed broader tissue tropism, including the ability to replicate in the upper and lower respiratory tract. This ability of H9N2 viruses to replicate in multiple compartments of the respiratory tract provides more opportunities for selecting transmissibility traits. These data further supports the notion that virulence of influenza viruses is a polygenic trait.

It is important to note that the avian H9N2 virus, RGWF10, and the H9N2 avian-human reassortant virus, which transmitted efficiently to the direct contact ferrets, failed to transmit to the aerosol contacts. Particularly, the ferrets infected with the reassortant virus displayed clinical signs, including sneezing, and shed virus titers similar to those observed for the parental H3N2 virus. Therefore the inability to transmit by aerosol cannot be attributed to lack of sufficient viral shedding or sneezing. It appears that avian H9N2 viruses, including those that have acquired SAα2-6 receptor specificity, still lack a key component necessary for efficient aerosol transmission among mammals and, perhaps, humans. It would be reasonable to speculate that the molecular restriction lies within the surface glycoproteins, particularly the HA. Despite this restriction in aerosol transmission, three key factors in avian H9N2 viruses should be noted. First, a number of studies have demonstrated that H9N2 viruses are undergoing extensive evolution and reassortment [3], [4], [6], [7] fueling their pandemic potential. Second, there have been several lines of evidence that H9N2 viruses have transmitted to pigs [5], [14]–[16], the proposed intermediate host that is permissive to both avian and human influenza viruses. The pig could therefore serve as an ideal environment for avian H9N2 viruses to acquire alterations favoring human infection and possibily human-to-human transmission. Third, serological data from separate studies suggest that there may be more human cases of H9N2 infection than previously anticipated [21], [23], and that the possibility of a limited level of human-to-human transmission cannot be absolutely excluded [20]. Therefore, avian H9N2 viruses are in an ideal position to undergo further adaptation for more efficient transmission among mammals and humans.

In summary, we have shown in this study that avian H9N2 viruses are able to replicate in the respiratory tract of ferrets and those viruses with Leu226 have propensity to transmit relatively efficiently to direct contacts. The transmission and replication phenotype can be further improved by providing the virus with a gene constellation more adapted for replication in ferrets. Efficient aerosol transmission, a prerequisite for a human pandemic, was not observed. However, considering the widespread prevalence of H9N2 viruses in poultry, the human virus-like receptor specificity of some avian and swine H9N2 isolates, co-circulation of H9N2 with H3N2 viruses in Asian swine, and the repeated direct transmission to humans, the public health threat of H9N2 viruses cannot be overemphasized. Further studies should aim at dissecting the molecular constraints that limit aerosol transmission of H9N2 viruses and the natural glycan profile of the mammalian respiratory tract.

## Materials and Methods

### Viruses

The wild-type (WT) viruses used in this study, including 8 avian H9N2 viruses, 1 avian H2N2 virus and a human H3N2 virus, are listed in Table 1. The viruses were kindly provided by Robert G. Webster from St. Jude Children's Research Hospital, Memphis, TN, and by Ilaria Capua from the OIE, FAO and National Reference Laboratory for Avian Influenza and Newcastle Disease, Padova, Italy. The recombinant A/Guinea fowl/Hong Kong/WF10/99 (H9N2) (RGWF10), A/Quail/Hong Kong/A28945/88 (H9N2) (RGQa88) and A/Memphis/14/98 (H3N2) (RGMemphis98) viruses, were recovered using reverse genetics as previously described [8], [46]. In addition, an H9N2 avian-human reassortant virus, 2WF10:6M98, which contains the hemagglutinin (HA) and neuraminidase (NA) genes of RGWF10 and the six internal genes of RGMemphis98, was also recovered. Briefly, the genes of RGWF10 and RGMemphis98 viruses were cloned using a set of universal primers described previously [8], [46]–[48]. Cloned genes were sequenced and compared to the corresponding viral sequences to confirm that the clones did not carry spurious mutations. Sequences were generated using the Big Dye Terminator v3.1 Cycle Sequencing kit 1 (Applied Biosystems, Foster City, CA) and a 3100 Genetic Analyzer (Applied Biosystems, Foster City, CA), according to the manufacturer's instructions. Recovery of the virus was verified by sequencing the ressortant's full-genome. All of the work and handling of this virus was performed in a USDA-approved biosafety level 3+ containment facility. Viruses were propagated in 10-day-old embryonated chicken eggs or Madin-Darby canine kidney (MDCK) cells. The median tissue culture infectious dose (TCID_50_) of each virus was determined in MDCK cells (primary chicken kidney cells were used for the RGQa88 and mQa88 due to increased susceptibility of these cells for these two viruses) [40].

### Site-directed mutagenesis

The QuickChange II site-directed mutagenesis kit (Stratagene, Inc., La Jolla, CA) was used to create specific mutations in the HA genes of avian H9N2 viruses. A leucine (Leu) to glutamine (Gln) mutation was introduced at amino acid position 226 in the HA of RGWF10, while the opposite mutation (Gln to Leu) was introduced at the same position in the HA of RGQa88. Mutant viruses were recovered, propagated, titrated, and sequenced as previously described [40].

### Glycan microarray analysis

Viruses were propagated in 9-day-old embryonated chicken eggs. After 48 h incubation at 35°C, the viruses were inactivated by 0.02% β-propiolactone (Sigma-Aldrich, St. Louis, MO). To confirm the allantoic fluid was no longer infectious it was blindly passed in eggs twice. Allantoic fluid containing inactivated viruses was clarified by low-speed centrifugation (1,000g, 5 minutes) and then concentrated by using Centricon Plus-70® centrifugal filters (Millipore, Billerica, MA). Concentrated, inactivated viruses were adjusted to a final concentration of 128 hemagglutination units per 50 µl in PBS containing 3% bovine serum albumin (BSA) and glycan microarray analysis was performed as previously described [42], [49], [50]. Briefly, after adsorption of virus, arrays were incubated with ferret anti-H9 antibody control serum in PBS-BSA (RGWF10, RGQa88, and their corresponding mutants mWF10 and mQa88), or sheep antisera to Hu/HK/1073/99 or goat antisera to Ck/HK/G9/97 (for Dk/HK/Y280/97) (all 1∶1000 dilution), followed by biotinylated anti-ferret, anti-sheep or anti-goat IgG, respectively. Bound antibodies were then detected with Alexa Flour 488 labeled streptavidin.

### Plaque assays

Viruses were examined by plaque assay in MDCK cells [51]. Briefly, confluent cell monolayers in 6-well plates were infected with 10-fold dilutions of virus in a total volume of 0.4 ml PBS for 1 h at 37°C. Cells were washed twice with PBS and covered with an overlay of modified Eagle's medium containing 0.9% agar, 0.02% BSA, 1% glutamine, and 1 µg/ml trypsin. The plates were then incubated at 37°C under 5% CO_2_. After 3 days of incubation the overlays were removed and the cells were stained with crystal violet.

### Infection and transmission in ferrets

Female Fitch ferrets, 3 to 6 months-old, were purchased from Triple F Farms (Sayre, PA). Prior to infection, ferrets were housed in a BSL2 facility and monitored for 5 to 7 days to measure body weight and establish baseline body temperatures. A subcutaneous implantable temperature transponder (Bio Medic Data Systems, Seaford, DE) was placed in each ferret for identification and temperature readings. Temperatures were recorded daily and fevers were defined as 3 standard deviations above the baseline reading. Three days before infection, blood was collected and serum tested for antibodies using the hemagglutination inhibition (HI) assay. Ferrets with HI titers at or lower than 10 were considered “influenza A-free” and were used in the study.

Ferret studies were performed in a BSL3+ facility in HEPA-filtered isolators. Studies were conducted under guidelines approved by the Animal Care and Use Committees of the University of Maryland and the Centers for Disease Control and Prevention. The basic set-up consisted of three ferrets: one infected, one direct contact and one aerosol contact. Ferrets were housed in wire cages placed inside isolators. Ferrets were lightly anesthetized with ketamine (20 mg/kg) and xylazine (1 mg/kg) via an intramuscular injection and inoculated intranasally (i.n.) with 10^6^ TCID_50_ of virus in PBS, 250 µl per nostril. Twenty-four hours later, two naïve ferrets were introduced into the isolator. One (direct contact) was introduced into the same cage as the infected ferret while the other (aerosol contact) was placed in a cage separated from the infected ferret by a wire mesh. The wire mesh prevented physical contact between the aerosol and infected/direct contact, allowing only air to be shared between the ferrets. All materials inside the cage of the inoculated ferrets were removed and replaced before introducing the direct contacts in order to ensure transmission occurred through contact with the inoculated ferrets and not infected/contaminated materials in the cage. Individual body temperatures and weights were measured daily. To monitor viral shedding, nasal washes were collected daily for up to two weeks. Briefly, ferrets were anesthetized as described above, and 1ml of PBS was used to induce sneezing. The nasal washes were collected into Petri dishes and brought to a total volume of 1ml with PBS. The nasal washes were immediately tested for virus using the FLU DETECT™ Antigen Capture Test Strip (Synbiotics Corp., San Diego, CA) and additional aliquots were stored at −80°C before performing TCID_50_ titration in MDCK cells. At day 14 pi and pc, blood was collected and seroconversion was determined by HI assay.

### HI assay

Serum samples were treated with receptor-destroying enzyme (Accurate Chemical and Scientific Corp., Westbury, NY) to remove nonspecific receptors and the anti-viral antibody titers were evaluated using the HI assay system outlined by the WHO Animal Influenza Training Manual (WHO/CDS/CSR/NCS/2002.5). HI assays were performed using homologous viruses as shown in the results section.

### Histopathology and tissue tropism

Groups of two ferrets were inoculated with 10^6^ TCID_50_ of each virus. Ferrets were euthanized on day 4 pi Brain, olfactory bulb, nasal turbinate, trachea, lung, heart and liver were collected and samples were both fixed with buffered neutral formalin for histological evaluation and stored at −80°C for virus titration. For histopathology, paraffin-embedded sections of 5-µm thickness were cut and stained with H&E (Histoserv, Inc., Germantown, MD). Representative microscopic photos were taken with the SPOT ADVANCED software (Version 4.0.8, Diagnostic Instruments, Inc., Sterling Heights, MI). To determine the tissue distribution of the virus, 10% (w/v) of tissue homogenate was prepared with PBS and the viral titers were determined in MDCK cells.
